# Supplementary material for: Deep divergence of Red-crowned Ant Tanager (Habia rubica: Cardinalidae), a multilocus phylogenetic analysis with emphasis in Mesoamerica
Source: PeerJ. 2018 Sep 12;6:e5496. doi: 10.7717/peerj.5496 (PMC6139011; doi:10.7717/peerj.5496)
Supplement: Table S1 — List of individuals sequenced, collection numbers, localities and georeferences. Museo de Zoología Alfonso L. Herrera (MZFC-UNAM); El Colegio de la frontera Sur, Unidad Chetumal (ECOSUR-CH); University of Washington, The Burke Museum (UWBM); (JK); Museum of Natural Science (Louisiana State University, LSUMNH) and The Natural History Museum (University of Kansas, KU). [file peerj-06-5496-s001.pdf]

#	Taxon (subspecies)	Collection number	Country	State/Province	Latitude	Longitude
1	*Habia rubica rosea*	MZFC URRA 55	México	Jalisco	20.4658	-105.2939
2	*Habia rubica rosea*	MZFC URRA 60	México	Jalisco	20.4658	-105.2939
3	*Habia rubica rosea*	MZFC URRA 63	México	Jalisco	20.4658	-105.2939
4	*Habia rubica rosea*	MZFC URRA 67	México	Jalisco	20.4658	-105.2939
5	*Habia rubica rosea*	MZFC CONACyT 799	México	Colima	19.4569	-103.7062
6	*Habia rubica rosea*	UWBM Michbts08126	México	Michoacán	18.0951	-102.3960
7	*Habia rubica rosea*	UWBM Michbts08196	México	Michoacán	18.1685	-102.3113
8	*Habia rubica rosea*	MZFC MOLGRO40	México	Guerrero	17.3579	-99.4625
9	*Habia rubica rosea*	MZFC MOLGRO41	México	Guerrero	17.3579	-99.4625
10	*Habia rubica rosea*	MZFC MOLGRO42	México	Guerrero	17.3579	-99.4625
11	*Habia rubica rosea*	MZFC MOLGRO43	México	Guerrero	17.3579	-99.4625
12	*Habia rubica rosea*	MZFC MOLGRO261	México	Guerrero	17.0038	-99.6437
13	*Habia rubica rosea*	MZFC MOLGRO262	México	Guerrero	17.0038	-99.6437
14	*Habia rubica rosea*	MZFC MOLGRO454	México	Guerrero	17.5303	-101.4428
15	*Habia rubica rosea*	MZFC MOLGRO455	México	Guerrero	17.5303	-101.4428
16	*Habia rubica rosea*	MZFC MOLGRO438	México	Guerrero	17.5303	-101.4428
17	*Habia rubica affinis*	MZFC OMVP160	México	Oaxaca	16.9633	-97.9067
18	*Habia rubica affinis*	MZFC OMVP161	México	Oaxaca	16.9633	-97.9067
19	*Habia rubica affinis*	MZFC OMVP672	México	Oaxaca	16.8283	-97.8800
20	*Habia rubica affinis*	MZFC OMVP682	México	Oaxaca	16.8200	-97.8883
21	*Habia rubica affinis*	MZFC OMVP1008	México	Oaxaca	16.2367	-97.2867
22	*Habia rubica affinis*	MZFC MIA81	México	Oaxaca	15.9250	-96.4200
23	*Habia rubica affinis*	UWBM Oaxjk07115	México	Oaxaca	16.1952	-97.1513
24	*Habia rubica affinis*	UWBM Oaxjk07058	México	Oaxaca	16.1952	-97.1513
25	*Habia rubica nelsoni*	MZFC Y408176	México	Campeche	18.0201	-90.3200
26	*Habia rubica nelsoni*	MZFC B2000	México	Campeche	18.5928	-90.2561
27	*Habia rubica nelsoni*	MZFC B2037	México	Campeche	18.5928	-90.2561
28	*Habia rubica nelsoni*	MZFC MOL1121	México	Campeche	18.5992	-89.2781
29	*Habia rubica nelsoni*	MZFC MOL1122	México	Campeche	18.5909	-89.2577
30	*Habia rubica nelsoni*	MZFC MOL1149	México	Quintana Roo	18.5992	-89.2781
31	*Habia rubica nelsoni*	ECOSUR-CH ADAB95289	México	Quintana Roo	19.1597	-87.8889
32	*Habia rubica holobrunnea*	MZFC Hgo-SLP143	México	Hidalgo	21.0750	-98.9550
33	*Habia rubica holobrunnea*	MZFC NAR28	México	Veracruz	18.7994	-96.9580
34	*Habia rubica holobrunnea*	MZFC TUX37	México	Veracruz	18.5888	-95.0980
35	*Habia rubica holobrunnea*	MZFC TXT15	México	Veracruz	18.5504	-95.1237
36	*Habia rubica holobrunnea*	MZFC TXT19	México	Veracruz	18.5504	-95.1237
37	*Habia rubica holobrunnea*	MZFC TXT58	México	Veracruz	18.3098	-94.8807
38	*Habia rubica holobrunnea*	MZFC TXT62	México	Veracruz	18.3062	-94.8798
39	*Habia rubica holobrunnea*	MZFC TXT63	México	Veracruz	18.3062	-94.8798
40	*Habia rubica holobrunnea*	MZFC TXT64	México	Veracruz	18.3008	-94.7216
41	*Habia rubica holobrunnea*	UWBM Ver343363	México	Veracruz	18.3211	-94.8319
42	*Habia rubica holobrunnea*	UWBM Ver393884	México	Veracruz	18.3211	-94.8319
43	*Habia rubica rubicoides*	MZFC CHIMA416	México	Oaxaca	17.0668	-94.0500
44	*Habia rubica rubicoides*	MZFC OMVP545	México	Oaxaca	17.0667	-94.5833
45	*Habia rubica rubicoides*	MZFC OMVP546	México	Oaxaca	17.0667	-94.5833
46	*Habia rubica rubicoides*	MZFC OMVP563	México	Oaxaca	17.0200	-94.6586
47	*Habia rubica rubicoides*	MZFC OMVP574	México	Oaxaca	17.0200	-94.6586
48	*Habia rubica rubicoides*	MZFC YACH378	México	Chiapas	16.0842	-90.9767
49	*Habia rubica rubicoides*	MZFC YACH528	México	Chiapas	16.9058	-90.9828
50	*Habia rubica rubicoides*	UWBM DHB4369	Guatemala	Quezaltenango	14.6500	-91.6000
51	*Habia rubica rubicoides*	JK DHB4362	Guatemala	Quezaltenango	14.6500	-91.6000
52	*Habia rubica rubicoides*	JK DHB4377	Guatemala	Quezaltenango	14.6500	-91.6000
53	*Habia rubica rubicoides*	JK DHB4399	Guatemala	Quezaltenango	14.6500	-91.6000
54	*Habia rubica rubicoides*	KU 5934	El Salvador	Ahuachapan	13.8100	-89.8070
55	*Habia rubica rubicoides*	KU 5937	El Salvador	Ahuachapan	13.8100	-89.8070
56	*Habia rubica rubicoides*	KU 5949	El Salvador	Ahuachapan	13.8100	-89.8070

57	*Habia rubica rubicoides*	KU 5974	El Salvador	Sonsonate	13.8280	-89.5670
58	*Habia rubica rubicoides*	KU 5978	El Salvador	Sonsonate	13.8280	-89.5670
59	*Habia rubica rubicoides*	KU 5982	El Salvador	Sonsonate	13.8280	-89.5670
60	*Habia rubica rubicoides*	KU 5985	El Salvador	Sonsonate	13.8280	-89.5670
61	*Habia rubica rubicoides*	JK 434136	El Salvador	Ahuachapan	13.9260	-89.8411
62	*Habia rubica rubicoides*	JK 434137	El Salvador	Sonsonate	13.6824	-89.6628
63	*Habia rubica rubicoides*	JK GAV2110	Honduras	La Ceiba	15.4300	-86.5200
64	*Habia rubica rubicoides*	JK GMS145	Honduras	La Ceiba	15.4300	-86.5200
65	*Habia rubica rubicoides*	JK EF529897	Honduras/ Nicaragua	ND	ND	ND
66	*Habia rubica rubicoides*	UWBM DAB1485	Nicaragua	Managua	11.9900	-86.2600
67	*Habia rubica rubicoides*	UWBM DAB1486	Nicaragua	Managua	11.9900	-86.2600
68	*Habia rubica rubicoides*	JK DAB1484	Nicaragua	Managua	11.9900	-86.2600
69	*Habia rubica rubicoides*	JK DAB1508, GMS1070	Nicaragua	Managua	11.9900	-86.2600
70	*Habia rubica rubicoides*	JK DAB1349	Nicaragua	Matagalpa	13.0150	-85.9240
71	*Habia rubica vinacea*	JK ZMUC130351	Costa Rica	Alajuela	10.3070	-84.8097
72	*Habia rubica vinacea*	JK GMS1113	Panamá	ND	8.6167	-80.1000
73	*Habia rubica vinacea*	JK GMS1013	Panamá	Santa Fe	8.0500	-81.1000
74	*Habia rubica vinacea*	UWBM JTK04160	Panamá	Santa Fe	8.0500	-81.1000
75	*Habia rubica vinacea*	JK GMS1070	Panamá	ND	8.6167	-80.1000
76	*Habia rubica vinacea*	JK JTK04138	Panamá	Santa Fe	8.0500	-81.1000
77	*Habia rubica vinacea*	JK JTK04166	Panamá	Santa Fe	8.0500	-81.1000
78	*Habia rubica vinacea*	JK ANSP5772	Panamá	ND	ND	ND
79	*Habia rubica vinacea*	JK ANSP5771	Panamá	ND	ND	ND
80	*Habia rubica rhodinolaema*	LSUMNH 27368	Perú	ND	-4.2325	-74.2179
81	*Habia rubica rhodinolaema*	LSUMNH 27369	Perú	Loreto	-4.2325	-74.2179
82	*Habia rubica rhodinolaema*	JK 40060	Perú	Loreto	-4.2325	-74.2179
83	*Habia rubica rhodinolaema*	JK 40061	Perú	Loreto	-4.2325	-74.2179
84	*Habia rubica rhodinolaema*	JK 457560	W Brasil	Amazonas	-1.6831	-65.8331
85	*Habia rubica rhodinolaema*	JK 457559	W Brasil	Amazonas	-1.8827	-66.9296
86	*Habia rubica rhodinolaema*	JK 457558	W Brasil	Amazonas	-2.4874	-68.2608
87	*Habia rubica hesterna*	JK ZMUC120394	W Brasil	Mato Grosso	-9.4478	-55.8570
88	*Habia rubica*	JK ANSP1495	Perú	ND	ND	ND
89	*Habia rubica peruviana*	JK 11166	Perú	Ucayali	-9.8251	-73.0878
90	*Habia rubica peruviana*	JK FMNH398440	Perú	Madre de Dios	-12.6669	-71.2706
91	*Habia rubica peruviana*	KU 457	Perú	Madre de Dios	-12.3300	-69.0300
92	*Habia rubica peruviana*	KU 628	Perú	Madre de Dios	-12.3300	-69.0300
93	*Habia rubica peruviana*	KU 636	Perú	Madre de Dios	-12.3300	-69.0300
94	*Habia rubica peruviana*	KU 711	Perú	Madre de Dios	-12.3300	-69.0300
95	*Habia rubica peruviana*	KU 722	Perú	Madre de Dios	-12.3300	-69.0300
96	*Habia rubica peruviana*	LSUMNH 22625	Bolivia	La Paz	-16.4997	-68.1500
97	*Habia rubica peruviana*	LSUMNH 8909	Bolivia	Pando	-10.7989	-66.9988
98	*Habia rubica peruviana*	LSUMNH 8959	Bolivia	Pando	-10.7989	-66.9988
99	*Habia rubica peruviana*	LSUMNH 12591	Bolivia	Santa Cruz	-17.8667	-63.0000
100	*Habia rubica peruviana*	LSUMNH 12594	Bolivia	Santa Cruz	-17.8667	-63.0000
101	*Habia rubica peruviana*	JK 1052	Bolivia	La Paz	-16.4997	-68.1500
102	*Habia rubica peruviana*	JK 22623	Bolivia	La Paz	-16.4997	-68.1500
103	*Habia rubica peruviana*	JK 18345	Bolivia	Santa Cruz	-17.8667	-63.0000
104	*Habia rubica peruviana*	JK ZMUC 145305	Bolivia	Beni	-14.3783	-65.0958
105	*Habia rubica peruviana*	LSUMNH 36646	W Brasil	Rondúnia	-11.5057	-63.5806
106	*Habia rubica rubica*	JK ZMUC137118	E Brasil	Sao Paulo	-23.6060	-46.4588
107	*Habia rubica rubica*	JK ZMUC137117	E Brasil	Sao Paulo	-23.6060	-46.4588
108	*Habia rubica rubica*	JK FMNH345472	E Brasil	Sao Paulo	-23.6060	-46.4588
109	*Habia rubica rubica*	KU 205	Paraguay	Caazapa	-26.3500	-55.5167
110	*Habia rubica rubica*	KU 226	Paraguay	Caazapa	-26.3500	-55.5167
111	*Habia rubica rubica*	KU 259	Paraguay	Caazapa	-26.3500	-55.5167
112	*Habia rubica rubica*	KU 313	Paraguay	Caazapa	-26.3500	-55.5167
113	*Habia rubica rubica*	KU 3662	Paraguay	Itapua	-26.5167	-55.8000
114	*Habia rubica rubica*	KU 3785	Paraguay	Itapua	-26.5167	-55.8000

115	*Habia rubica rubica*	KU 3852	Paraguay	Itapua	-26.5167	-55.8000
116	*Habia rubica rubica*	KU 3853	Paraguay	Itapua	-26.5167	-55.8000
117	*Habia rubica rubica*	LSUMNH 25853	Paraguay	Caazapá	-26.2261	-56.0250
118	*Habia rubica rubica*	JK 25909	Paraguay	Caaguazú	-25.4600	-56.0200
119	*Habia rubica rubica*	JK 25830	Paraguay	Caazapá	-26.2261	-56.0250
120	*Habia rubica rubica*	JK ZMUC144784	Paraguay	Caazapá	-26.0667	-55.7500
121	*Habia rubica rubica*	JK MVZ168909	Paraguay	Itapúa	-27.1833	-55.7778
122	*Habia rubica rubica*	UWBM DHB1801	Argentina	Misiones	-26.9552	-55.0877
123	*Habia rubica rubica*	UWBM GAV821	Argentina	Misiones	-26.9552	-55.0877
124	*Habia rubica rubica*	UWBM GAV822	Argentina	Misiones	-26.9552	-55.0877
125	*Habia rubica perijana*	JK IC 1110	Venezuela	Barinas	8.3137	-70.0503
126	*Chlorothraupis carmioli*	LSUMNH 106762	Bolivia	Beni	-14.3783	-65.0958
127	*Chlorothraupis carmioli*	LSUMNH 1068	Bolivia	La Paz	-16.4997	-68.1500
128	*Chlorothraupis carmioli*	LSUMNH 27849	Perú	Loreto	-4.2325	-74.2179
129	*Chlorothraupis carmioli*	LSUMNH 28343	Panamá	Panamá	8.9831	-79.5167
130	*Chlorothraupis carmioli*	LSUMNH 2060	Perú	Pasco	-10.4476	-75.1545
131	*Chlorothraupis carmioli*	LSUMNH 21207	Perú	Puno	-15.8402	-70.0219
132	*Chlorothraupis carmioli*	LSUMNH 5510	Perú	San Martín	-7.2445	-76.8260
133	*Chlorothraupis carmioli*	GB EF529900	Ecuador	ND	ND	ND
134	*Chlorothraupis carmioli*	GB KC007580	Perú	San Martín	-7.2445	-76.8260
135	*Chlorothraupis olivacea*	LSUMNH 2196	Panamá	Darién	7.8682	-77.8367
136	*Chlorothraupis olivacea*	LSUMNH 52930	Panamá	Darién	7.8682	-77.8367
137	*Chlorothraupis olivacea*	GB EF529901	Ecuador	ND	ND	ND
138	*Chlorothraupis stolzmanni*	LSUMNH 7860	Ecuador	El Oro	-3.4455	-79.8297
139	*Chlorothraupis stolzmanni*	LSUMNH 7876	Ecuador	El Oro	-3.4455	-79.8297
140	*Chlorothraupis stolzmanni*	LSUMNH 11847	Ecuador	Esmeraldas	-0.9682	-79.6517
141	*Chlorothraupis stolzmanni*	GB EF529899	Ecuador	ND	-2.9650	-79.1107
142	*Habia gutturalis*	JK TA15	Colombia	ND	ND	ND
143	*Habia atrimaxillaris*	UWBM ND	Costa Rica	ND	ND	ND
144	*Habia fuscicauda*	MZFC B1908	México	Campeche	18.5928	-90.2360

List of individuals sequenced, collection numbers, localities and georeferences. Museo de Zoología Alfonso L. Herrera (MZFC-UNAM); El Colegio de la frontera Sur, Unidad Chetumal (ECOSUR-CH); University of Washington, The Burke Museum (UWBM); (JK); Museum of Natural Science (Louisiana State University, LSUMNH) and The Natural History Museum (University of Kansas, KU).
