# Supplementary material for: Deep divergence of Red-crowned Ant Tanager (Habia rubica: Cardinalidae), a multilocus phylogenetic analysis with emphasis in Mesoamerica
Source: PeerJ. 2018 Sep 12;6:e5496. doi: 10.7717/peerj.5496 (PMC6139011; doi:10.7717/peerj.5496)
Supplement: Table S2 — Genetic diversity by phylogroups from mitochondrial DNA phylogeny. Numbers of individuals (n), haplotypes (H), segregation sites (s), haplotype diversity (Hd) nucleotide diversity (π), Tajima and Fu parameters. [file peerj-06-5496-s002.pdf]

Mitochondrial phylogroups		*n*	*H*	*S*	*Hd*	π	Tajima's *D*		Fu's *Fs*	
							D	P	*Fs*	P
MX West Pacific	(A)	5	2	1	0.4	0.00044	-0.8165	P>0.10	-0.8165	P>0.10
MX South Pacific	(B)	19	2	1	0.28070	0.00031	-0.77799	P>0.10	-0.5736	P>0.10
Eastern MX, Gulf Coast	(C)	16	5	3	0.73333	0.00108	-0.34679	P>0.10	-0.7063	P>0.10
Yucatan Peninsula to Costa Rica	(D)	18	6	10	0.82353	0.00414	0.33984	P>0.10	0.45503	P>0.10
Guatemala and El Salvador	(d)	13	4	3	0.52564	0.00065	-1.43714	P>0.10	-1.35316	P>0.10
Panama	(E)	8	5	9	0.85714	0.00395	-0.04534	P>0.10	0.139	P>0.10
Western South America	(F)	26	17	24	0.93538	0.00311	-2.20278	**P<0.01	-3.20159	**P<0.02
Eastern South America	(G)	20	8	38	0.83684	0.00646	-2.06341	*P<0.05	-3.27526	**P<0.02

Genetic diversity by phylogroups from mitochondrial DNA phylogeny. Numbers of individuals (*n*), haplotypes (*H*), segregation sites (*s*), haplotype diversity (*Hd*) nucleotide diversity (π), Tajima and Fu parameters.
