# Supplementary figures and images for: Deep divergence of Red-crowned Ant Tanager (Habia rubica: Cardinalidae), a multilocus phylogenetic analysis with emphasis in Mesoamerica
Source: PeerJ. 2018 Sep 12;6:e5496. doi: 10.7717/peerj.5496 (PMC6139011; doi:10.7717/peerj.5496)

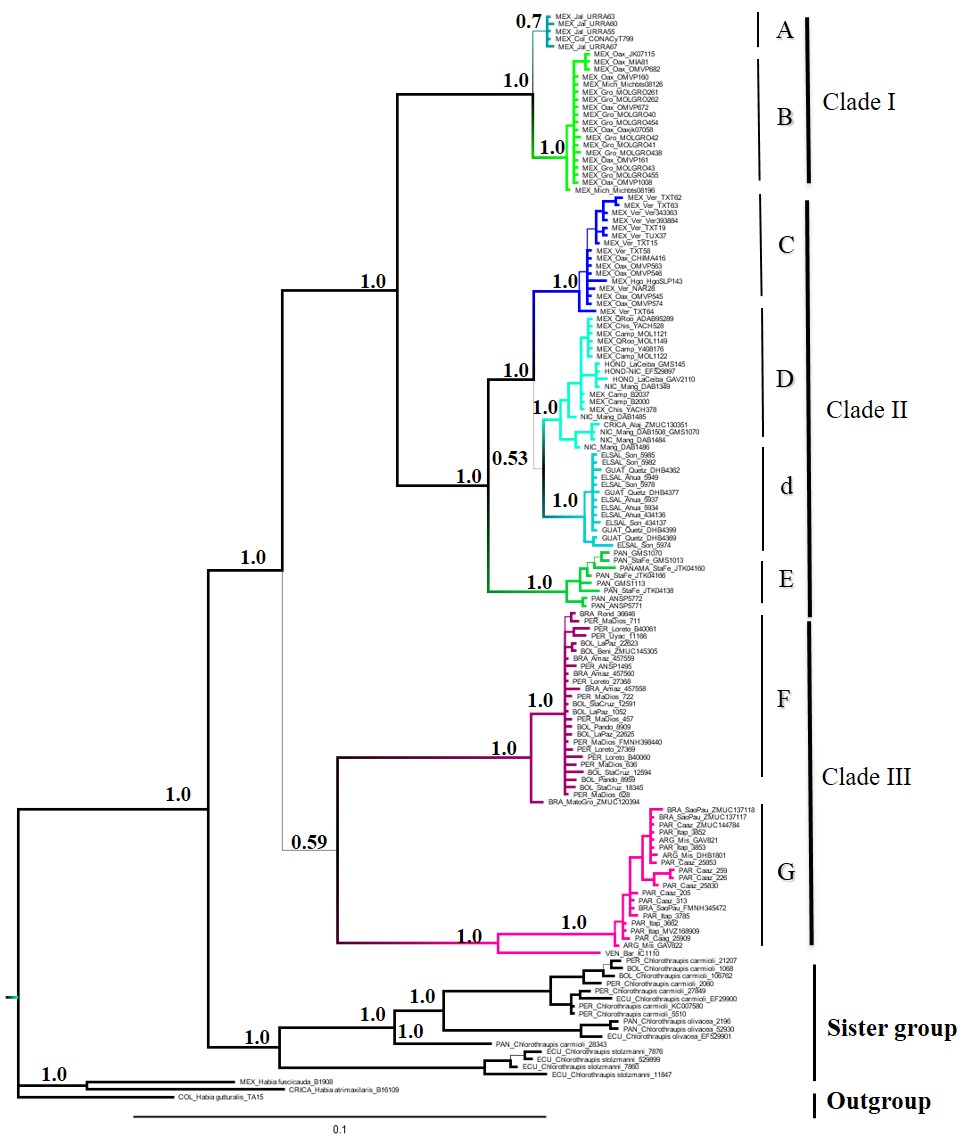

Supplement: Figure S1 — Phylogenetic consensus tree representing the relationships among Habia rubica populations based on Bayesian inference from mitochondrial dataset. Values above branches denote posterior probabilities. Thin branches of the tree denote low values of posterior probabilities (PP < 0.6). [file peerj-06-5496-s003.jpg]
